# Characterization of drought stress-mitigating *Rhizobium* from faba bean (*Vicia faba* L.) in the Chinese Qinghai-Tibet Plateau

**DOI:** 10.3389/fmicb.2023.1212996

**Published:** 2023-08-24

**Authors:** Ping Li, Changcai Teng, Jinfa Zhang, Yujiao Liu, Xuexia Wu, Tao He

**Affiliations:** ^1^Academy of Agriculture and Forestry Sciences of Qinghai University, Qinghai Academy of Agriculture and Forestry Sciences, Xining, Qinghai, China; ^2^State Key Laboratory of Plateau Ecology and Agriculture, Qinghai University, Xining, Qinghai, China; ^3^College of Agriculture and Animal Husbandry, Qinghai University, Xining, Qinghai, China

**Keywords:** *Rhizobium*, phylogeny, biochemical characterization, drought tolerance, *Vicia faba*

## Abstract

*Rhizobium*-driven symbiotic nitrogen-fixation in legumes not only benefits the growth but also enhances the stress tolerance of plants. Isolating and characterizing efficient, drought-tolerant rhizobia is a central goal for improving crop yields in arid regions. Here, we phylogenetically and biochemically characterized a novel strain of *Rhizobium* (‘QHCD11’) sampled from the root nodules of faba beans growing in an arid agricultural area in Qinghai-Tibet. We further tested the drought tolerance of the strain as well as of ‘Qingcan 14’ faba bean seedlings inoculated with it. Biochemical characterization involved bromothymol blue (BTB) tests, carbon metabolic profiling (Biolog GENIII), DNA–DNA hybridization (dDDH) assays, average nucleotide identity (ANI) analyses, and 16S rRNA sequencing. The result indicated that strain ‘QHCD11’ likely belongs to the *Rhizobium indicum* species. Drought stress tolerance was assessed by exposure to polyethylene glycol (PEG-6000) at concentrations of 0, 10, 15, and 20%. Increasing concentrations of PEG-6000 tended to result in decreased growth of ‘QHCD11’, although the strain performed better at 20% PEG 6000 than at 15%. Inoculation of drought-stressed faba bean seedlings with strain ‘QHCD11’ improved root vitality, chlorophyll content, antioxidant enzyme activities, and plant height. We suggest that inoculation of faba beans with ‘QHCD11’ is an environmentally sound strategy for mitigating crop drought stress in arid and semi-arid regions. In addition, the results presents here will benefit future studies into faba bean-rhizobia symbioses under drought stress.

## Introduction

Nitrogen (N) is arguably one of the most important in all of the essential plant nutrients. Globally, the nitrogen cycle transforms approximately 3 × 10^9^ tons of gaseous N_2_ into plant- available forms annually. Importantly, approximately 60% of the total N conversion results from biochemical activities (Hardarson, 2003; Mabrouk and Belhadj, 2010). *Rhizobium*, a genus of Gram-negative soil bacteria, can develop symbiotic relationships with leguminous plants in the form of root nodules, they fix atmospheric nitrogen (Remigi et al., 2016; Stefan et al., 2018). As one of the most important contributors to total biological nitrogen fixation, *Rhizobium*-legume symbioses contribute more than 50% of the nitrogen available in agricultural soils (Herridge et al., 2008). Not only does this symbiotic relationship enhance nitrogen fixation capacity, but it also improves legume productivity and yield (Jida and Assefa, 2012; Laranjo et al., 2014; Alinia et al., 2022).

*Rhizobium* collected from the nodules and roots of legumes may be promising biofertilizers for use in improving crop growth, nodulation, nitrogen fixation, and resistance to stress and disease. Several studies indicate that naturally- occurring populations of rhizobia from certain geographic regions exhibit greater nodulation competency than many commercial strains (Gaci et al., 2021; Tuan Vo et al., 2021). The isolation and characterization of native rhizobial populations is likely to provide valuable biological resources for the enhancement of crop productivity (Humaira and Bano, 2011). Several studies have subjected native rhizobial populations isolated from legume crops to molecular identification through the use of genomic sequence-based digital DNA–DNA hybridization (dDDH), average nucleotide identity (ANI) analysis, and 16S rRNA sequencing (Deng et al., 2022; Zaw et al., 2022). It is clear from these studies that phylogenetic analysis is a stable and dependable method for the description and classification of species.

Unfavorable environmental conditions can compromise the legume-*Rhizobium* relationship, including drought and excessively high temperatures. Because of this, it is crucial to study legume-*Rhizobium* symbioses under such conditions. Studies suggest that host plants are often less tolerant of environmental stress than their microbial partners (Jebara et al., 2001; Mnasri et al., 2007; Vriezen et al., 2007). Inoculating seeds with *Rhizobium* has been shown to enhance the environmental stress tolerance of plants (Franzini et al., 2013; Zeenat et al., 2021). Such increased resistance likely results from a combination of decreased generation of reactive oxygen species (ROS), improvement of leaf water status, facilitation of water uptake, mitigation of ionic imbalance, and more efficient nutrient acquisition. Furthermore, utilizing highly efficient and stress-tolerant rhizobial inocula can further enhance the yield of legume crops in unfavorable environments (Peoples et al., 2009; Denton et al., 2017; Kanonge-Mafaune et al., 2018). Rhizobia appear to boost the stress resilience and growth of host plants through several mechanisms, including inducing the biosynthesis of osmolytes such as ectoines, polyamines, proline, and glycine betaine to guard cells from osmotic stress- and desiccation-related damage; promoting the activity of both enzymatic and non-enzymatic antioxidants; and increasing photosynthetic activity and biomass accumulation (Reina-Bueno et al., 2012; Wdowiak-Wróbel et al., 2013; Lunn et al., 2014; Ding, 2022). Taken together, it is clear that rhizobia can support plant health in unfavorable conditions. This ability may be crucial for enhancing legume cultivation on marginal land and in semi-arid and arid regions.

Qinghai is situated in northwestern China, in the northeastern section of the Qinghai-Tibet Plateau, and has an average altitude of more than 3,000 m. Seventy percent of the agricultural production of Qinghai occurs in semi-arid and arid regions (Li et al., 2018). Faba bean (*Vicia faba* L.; Fabaceae) has proven adaptable to the varied climates of this region, and is utilized as a grain, vegetable, and fodder crop. In addition, this leguminous crop enhances the sustainability of local cropping systems by contributing nitrogen to agricultural fields through biological fixation (Amede et al., 1999; Khan et al., 2007). At present, faba bean is the primary legume crop grown across the Qinghai-Tibet Plateau, and is a major contributor to economic and ecological development in the region. Unfortunately, faba bean is considerably less drought tolerant than other legumes such as chickpea, pea, and common bean (McDonald and Paulsen, 1997; Amede and Schubert, 2003; Sharma et al., 2017).

Here, we sought to determine the phylogenetic status and symbiotic efficacy of the *Rhizobium* strain ‘QHCD11’, which was isolated from an arid agricultural area in Qinghai-Tibet. We analyzed the morphological, physiological, biochemical, and genetic characteristics of *Rhizobium* strain ‘QHCD11’, as well as assessed its ability to colonize and enhance the drought resistance and growth of faba beans. The results of this study help to illuminate the complex relationship between rhizobia and legumes under stressful environmental conditions. We suggest that *Rhizobium* strain ‘QHCD11’ may be further developed into a useful inoculant for improving the drought tolerance of faba beans.

## Materials and methods

### Root nodule

The root nodule was obtained from an arid agriculture area in Haidong City, Minhe County, Qinghai Province, China (36.0607° N, 102.4520° E). The field soil pH was 8.1, with 16.65 g kg^−1^ organic matter (OM), 1.20 g kg^−1^ total N, 82 mg kg^−1^ alkaline N, 2.11 g kg^−1^ total phosphorus (P; P_2_O_5_), 24.6 mg kg^−1^ available P, 20.65 g kg^−1^ total potassium (K; K_2_O), 178 mg kg^−1^ available K, and 0.62 g kg^−1^ total salt. A single healthy plant at flowering stage was uprooted along with nodules and transported to the laboratory. The nodules was chose to isolate of red color (leghemoglobins).

### Isolation of rhizobial cultures

Faba bean root nodules were isolated in accordance with the method of Vincent (1970). The *Rhizobium* isolate was grown on yeast mannitol agar (YMA) plates at 28°C. The pure culture was preserved in 10% (v/v) glycerol stock for subsequent analyses. All morphological, biochemical, and molecular studies were carried out as described previously (Li et al., 2021). The reference *Rhizobium leguminosarum* strain ‘ACCC15854’ was obtained from the Agriculture Culture Collection of China (ACCC; Beijing, China).

### BTB test of *Rhizobium* ‘QHCD11’

To determine the alkali and acid production of the pure culture isolates, they were streaked onto fresh YMA plates (pH 6.8) containing 30 μg L^−1^ bromothymol blue (BTB) (Sharma et al., 2010). In a slight alteration of the original method, all plates were incubated for 3–5 d at 28°C, and the plates were evaluated each day. When acidic compounds are produced, the plate color changes from green to yellow, and when alkaline compounds are produced, the plate color changes from green to blue.

### Biochemical evaluation of *Rhizobium* ‘QHCD11’

To evaluate the biochemical and metabolic profile of *Rhizobium* strain ‘QHCD11’, we studied the biochemical parameters associated with carbon metabolism. A Biolog GEN III MIDI system (BIOLOG, CA, United States) was used to test whether *Rhizobium* strain ‘QHCD11’ could utilize 95 different carbon sources, according to the manufacturer’s instructions. Briefly, the isolate was cultured for 24 h in YM broth. The cells were then purified twice by 3 min of centrifugation at 8000 rpm. Subsequently, the cells were resuspended in sterilized water and adjusted to 10^8^ cells ml^−1^. Both experimental and control samples were prepared according to the manufacturer’s standard protocol. Positive (+) tests were indicated by a color change to purple, and a negative (−) tests were indicated by a lack of color change.

### 16S rRNA gene sequencing

To amplify the 16S rRNA gene cluster, the 16S-27F (5’-GTTTGATCM- TGGCTCAG-3′) and 16S-1492R (5’-TACGGYTACCTTGTTACGACTT-3′) primers were used, as described by Chelius and Triplett (2001). Each 25 μL PCR reaction mixture contained 3 μL of DNA template, 1 μL each of 16S rRNA reverse and forward primers, 12.5 μL of PCR mix, and 7.5 μL of double-distilled water (DDW). The Ezbiocloud database^1^ was used to search the partial 16S rRNA gene. A neighbor-joining (NJ) tree was constructed, using 1,000 bootstrap replications, in in MEGA 7.0. Kimura’s two-parameter model was utilized to determine distances (Kimura, 1980).

### Genomic characteristics of *Rhizobium* ‘QHCD11’

After the removal of connector sequences, the sequencing data were assembled to construct contigs and scaffolds using A5-MiSeq and SPAdes. The assemblies were evaluated and compared, and base correction was performed, using Pilon. Subsequent to filtration, PGCGAP was utilized to assemble the high-quality paired-end reads (Liu et al., 2021). Genomic reannotation was performed with Prokka (Seemann, 2014). Using the draft genome, the RAST server was utilized to determine the G + C content (Brettin et al., 2015). Both the ANI and dDDH were utilized to determine the taxonomic status of the novel isolate. JSpecies WS^2^ was used to obtain ANI values and the Genome-to-Genome Distance Calculator (GGDC)^3^ was used to obtain dDDH values.

### Drought tolerance of *Rhizobium* ‘QHCD11’

The drought tolerance of *Rhizobium* ‘QHCD11’ was assessed by culturing the isolate in YMA liquid culture medium supplemented with polyethylene glycol (PEG-6000) at 0, 10, 15%, or 20% (w/v). Each liquid culture vial was inoculated with 0.1 mL of standard bacterial suspension and each experiment was replicated three times. The tubes were incubated for 72 h at 28 ± 2°C in an orbital shaker at 160 rpm. Growth was assessed using a spectrophotometer to observe the optical densities at 600 nm (OD_600nm_), with PEG-free YMA liquid culture medium used as the blank. For comparison, the *R. leguminosarum* reference strain ‘ACCC15854’ was also tested.

### Inoculation of faba beans with *Rhizobium*

Faba bean‘Qingcan 14’ seeds were provided by the Qinghai Academy of Agriculture and Forestry Science (Xining City, Qinghai Provence, China). The surfaces of healthy, uniform seeds were sterilized using 1% sodium hypochlorite and washed at least three times with sterilized water. The seeds were subsequently germinated in darkness at 28°C in a petri dish carrying wet filter paper. The stored strain QHCD11 with 10% glycerol was streaked onto Yeast Mannitol Agar (YMA) plates and incubated at 28°C for 3 to 5 days until good growth was observed. A standard curve describing the relationship between cell number and optical density at 600 nm (OD_600nm_), was developed for strain QHCD11 to enable the application of a standard CFU mL^−1^ across experiments. Moreover, population counts (CFU mL^−1^) were determined by dilution plating. One milliliter of rhizobial suspension (≈10^8^ CFU mL^−1^) was used to inoculate each test tube containing 20 mL of YMB media. When the radicle reached 0.5–1.0 cm, seedlings were soaked in a *Rhizobium* standard suspension (10^8^ CFU mL^−1^) for 30 min and then planted in sterile containers containing quartz sand. To the base of each 5-day-old seedling was added an additional 2 mL of inoculum (10^8^ CFU mL^−1^). Each week, seedlings were provided with 20 mL of sterilized water and 20 mL of N-free McKnight’s nutrient solution. The experiment treatments consisted of four different treatments, no inoculation plants under well-watered conditions (NN-CK), no inoculation drought-stressed plants (NN-DS), inoculation plants under well-watered conditions (NA-CK), and inoculation plants under drought stress (NA-DS). Three replicates and three pots per replicate were designated for each treatment, making a total of 36 pots. All plants were grown in a greenhouse with natural daylight from 9 March to 9 Jun. The average day/night temperature was 26/20°C, air relative humidity was 70–80%. The plants were irrigated with distilled water every 1–2 days, and supplied with ½-strength Hoagland’s nutrient solution every 30 days (approximately 200 mL per pot). Drought stress was applied during faba bean flowerings stage and was subjected to withdrawing water for 3 days. Physiological index measurements were performed on plants after being subjected to drought stress for 3 days. As a positive control, additional seedlings were inoculated with the *R. leguminosarum* reference strain ‘ACCC15854’, while uninoculated seedlings were used as negative controls. All treatments were arranged in a randomized block design, and included uninoculated seedlings under well-watered conditions (NN-CK), uninoculated seedlings under drought conditions (NN-DS), inoculated seedlings under well-watered conditions (NA-CK), and inoculated seedlings under drought conditions (NA-DS). Each treatment consisted of three replicates, each replicate consisted of three pots, and a total of 36 pots were used for the experiment. All seedlings were cultivated in a climate-controlled chamber with 70–80% relative humidity and an average night/day temperature of 20/25°C. All morphological, physiological, and biochemical studies were carried out during the blooming stage. The tested indices included root mitochondrial respiration [triphenyltetrazolium chloride (TTC) method], superoxide dismutase (SOD) activity, proline (PRO) content, leaf relative water content (RWC), chlorophyll content (SPAD), root length, fresh root nodule weight, number of root nodules, and plant height. The root mitochondrial respiration (TTC), superoxide dismutase (SOD) and proline (PRO) content were determined using a TTC Assay Kit, a SOD Assay Kit and a PRO Assay Kit, respectively (Nanjing Jiancheng Bioengineering Institute, China). SPAD was evaluated with a SPAD- 502 meter (Konica Minolta, Japan). As described by Mayak et al. (2004), RWC (%) was calculated as [(FW-DW)/ (FTW-DW)] × 100, where FTW is the fully turgid weight, DW the dry weight, and FW is the fresh weight.

### Statistical analysis

Statistically significant differences were determined using the standard error (SE) of at least three replicates with SPSS 17.0 (SPSS, IL, United States).

## Results

### Characterization of *Rhizobium* isolated from faba bean plants

The isolate was purified by repeated plate streaking, and cultured on YMA plates at 28°C for 3 d in darkness. Morphologically, the isolates were described as 3–5 mm diameter, mucilaginous, opaque, shiny white colonies with a wet surface, which produced copious amounts of sticky exopolysaccharides. Microscopically, the bacteria were Gram-negative, rot-shaped, and red in color, which matches the general description of *Rhizobium.* The YMA-BTB-based acid/alkali production test indicated that the isolate was producing acidic compounds (green to yellow plate color transition) in medium. These result suggested that the isolate was a species of *Rhizobium*.

### Phylogenetic analysis and genomic characteristics of the *Rhizobium* isolate

**Figure 1:**
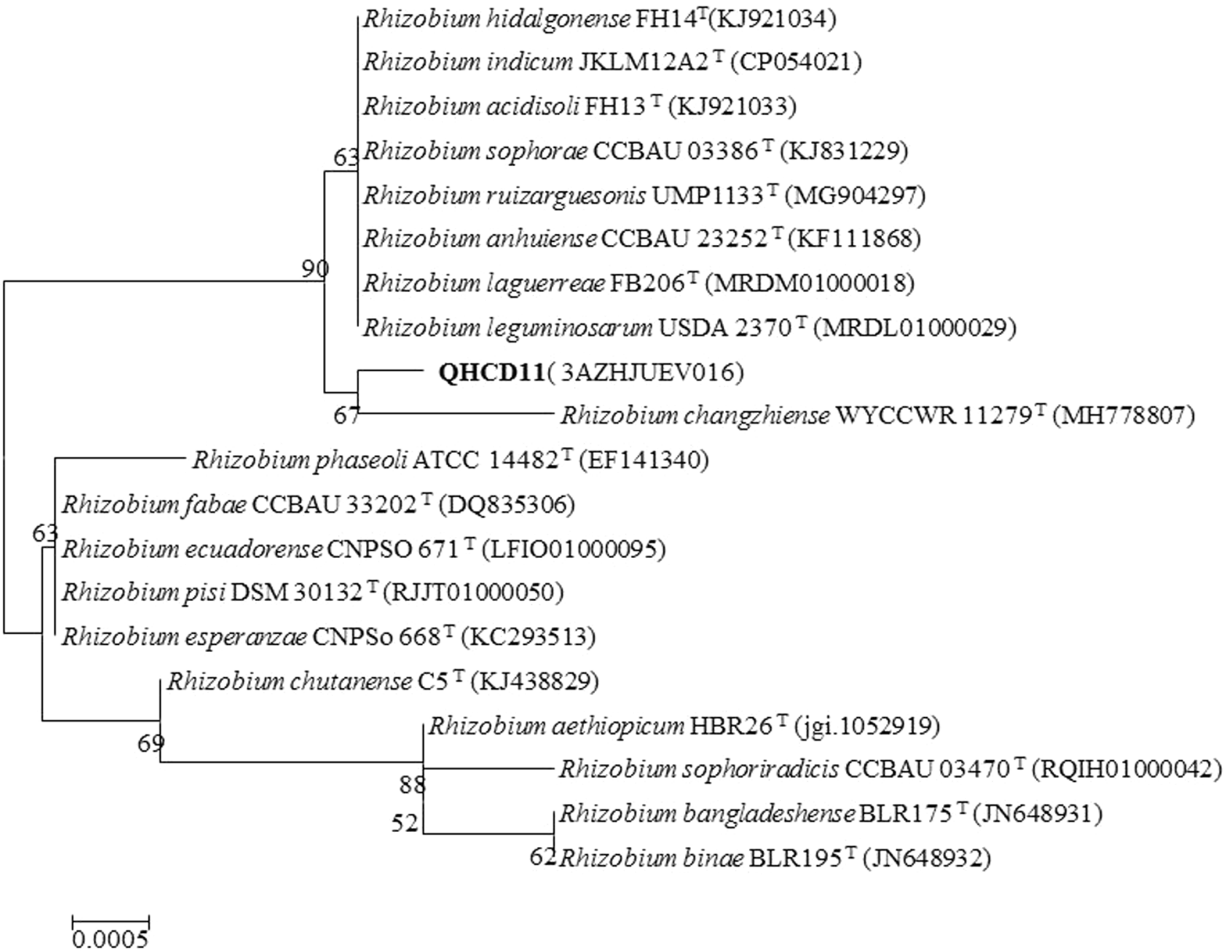
Neighbor-joining (NJ) phylogenetic analysis of *Rhizobium* ‘QHCD11’ near-full- length 16S rRNA gene sequences. Branch points show bootstrap values >50%. Scale = 0.0005 nucleotide substitutions per position.

The complete nucleotide sequence (1,477 bp) of the 16S rRNA gene was evaluated. An EzBioCloud sequence search revealed that strain ‘QHCD11’ shared 99.93% similarity were the *Rhizobium* type strains *R. leguminosarum* ‘USDA 2370’^T^, *R. anhuiense* ‘CCBAU 23252’^T^, *R. laguerreae* ‘FB206’^T^, *R. ruizarguesonis* ‘UMP1133’^T^, and *R. indicum* ‘JKLM12A2’^T^ (Table 1). The constructed NJ phylogenetic tree indicated that *Rhizobium* ‘QHCD11’ formed a phyletic group with *R. changzhiense* ‘WYCCWR 11279’^T^ (MH778807) (Figure 1). Our results were largely in agreement with previous studies suggesting that variation between Rhizobial strains may be more common at the species level, with diversity observed genus-wide.

**Table 1:** 16S rRNA gene sequence comparative analysis.

Rank	Species	Strain Name	Accession #	Pairwise Similarity (%)	Mismatch/ Total nt
1	*R. leguminosarum*	USDA 2370	MRDL01000029	99.93	1/1338
2	*R. laguerreae*	FB206	MRDM01000018	99.93	1/1338
3	*R. anhuiense*	CCBAU 23252	KF111868	99.93	1/1338
4	*R. ruizarguesonis*	UMP1133	MG904297	99.93	1/1338
5	*R.indicum*	JKLM12A2	CP054021	99.93	1/1337
6	*R. sophorae*	CCBAU03386	KJ831229	99.92	1/1297
7	*R. acidisoli*	FH13	KJ921033	99.92	1/1291
8	*R. hidalgonense*	FH14	KJ921034	99.92	1/1291
9	*R. changzhiense*	WYCCWR11279	MH778807	99.83	2/1210
10	*R.esperanzae*	CNPSo 668	KC293513	99.60	5/1236
11	*R. phaseoli*	ATCC 14482	EF141340	99.25	10/1338
12	*R. pisi*	DSM 30132	RJJT01000050	99.25	10/1338
13	*R. ecuadorense*	CNPSO 671	LFIO01000095	99.25	10/1338
14	*R. fabae*	CCBAU 33202	DQ835306	99.24	10/1312
15	*R. chutanense*	C5	KJ438829	99.23	10/1297
16	*R. sophoriradicis*	CCBAU 03470	RQIH01000042	99.10	12/1338
17	*R. aethiopicum*	HBR26	jgi.1052919	99.10	12/1338
18	*R. bangladeshense*	BLR175	JN648931	99.03	13/1338
19	*R. binae*	BLR195	JN648932	99.03	13/1338
20	*R. aegyptiacum*	1010	JQ670243	99.03	13/1338

**Figure 2:**
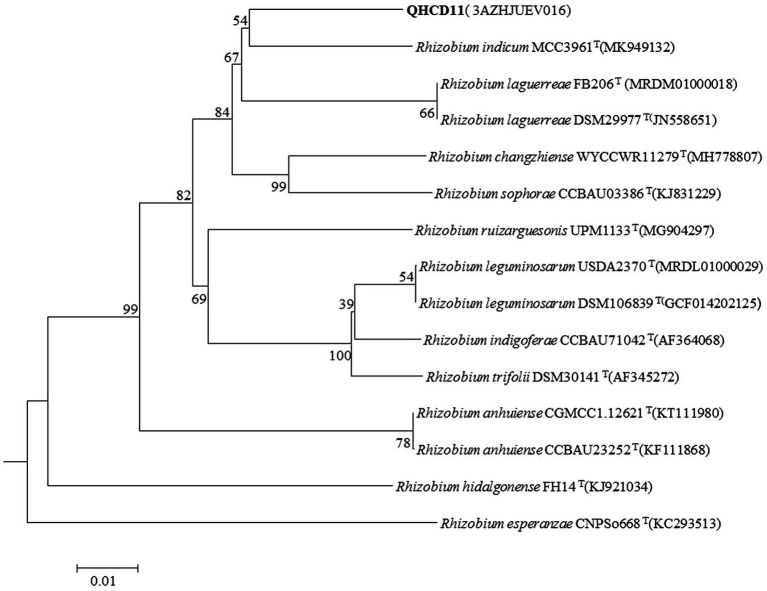
Neighbor-joining (NJ) phylogenetic analysis of *Rhizobium* ‘QHCD11’ whole genome sequence. Scale = 0.01 nucleotide substitutions per position. The outgroup was *R. esperanzae* ‘CNPSo668’^T^.

The draft genome of *Rhizobium* ‘QHCD11’ was 7.50 Mbp in size and the G + C content was 60.9%. The ANI value of *Rhizobium* ‘QHCD11’ was 95.62%, which was the same as that of *R. indicum* ‘FA23’^T^ (Table 2). To confirm the taxonomic status, dDDH values were also compared between strains (Tindall et al. 2010). The dDDH value of *Rhizobium* ‘QHCD11’ was 69.7%, indicating that it is a species of *R. indicum* ‘MCC 3961’^T^ (Table 3). Furthermore, in the phylogenomic tree, strain ‘QHCD11’ clustered with *R. indicum* ‘MCC 3961’^T^ (Figure 2).

**Table 2:** ANI analysis (%).

	11	1	2	3	4	5	6	7	8
11	100	91.66	93	94.35	93.64	89.18	88.06	**95.62**	88.33
		100	91.6	90.99	91.4	89.35	88.25	91.23	88.43
			100	92.52	93.51	89.19	88.12	93.08	88.29
				100	93	89.05	87.98	94.56	88.22
					100	89.26	88.15	93.72	88.33
						100	88.55	90	89.28
							100	88.36	89.73
								100	89.13
									100

1. *R. anhuiense* ‘CCBAU 23252’^T^; 2. *R. leguminosarum* ‘USDA 2370’^T^; 3. *R. laguerreae* ‘FB206’^T^; 4. *R. ruizarguesonis* ‘GB30’^T^; 5. *R. hidalgonense* ‘FH14’^T^; 6. *R. esperanzae* ‘CNPSo 668’^T^; 7. *R. indicum* ‘FA23’^T^; 8. *R. phaseoli* ‘ATCC 14482’^T^. The meaning of the bold values is which the ANI value of *Rhizobium* ‘QHCD11’ was 95.62%, which was the same as that of *R. indicum* ‘FA23’T in table.

**Table 3:** dDDH analysis (%).

Strain	*Rhizobium indicum* ‘MCC 3961’^T^	*Rhizobium laguerreae* ‘DSM 29977’^T^	*Rhizobium changzhiense ‘*WYCCWR 11279T’^T^	*Rhizobium leguminosarum ‘*USDA 2370’^T^	*Rhizobium sophorae ‘*CCBAU 03386’^T^
QHCD11	**69.7**	64.1	65	58.4	65.3

The meaning of the bold values is which the dDDH value of *Rhizobium* ‘QHCD11’ was 69.7%, indicating that it is a species of *R. indicum* ‘MCC 3961’T in table.

### Chemotaxonomic characteristics of the *Rhizobium* isolate

**Figure 3:**
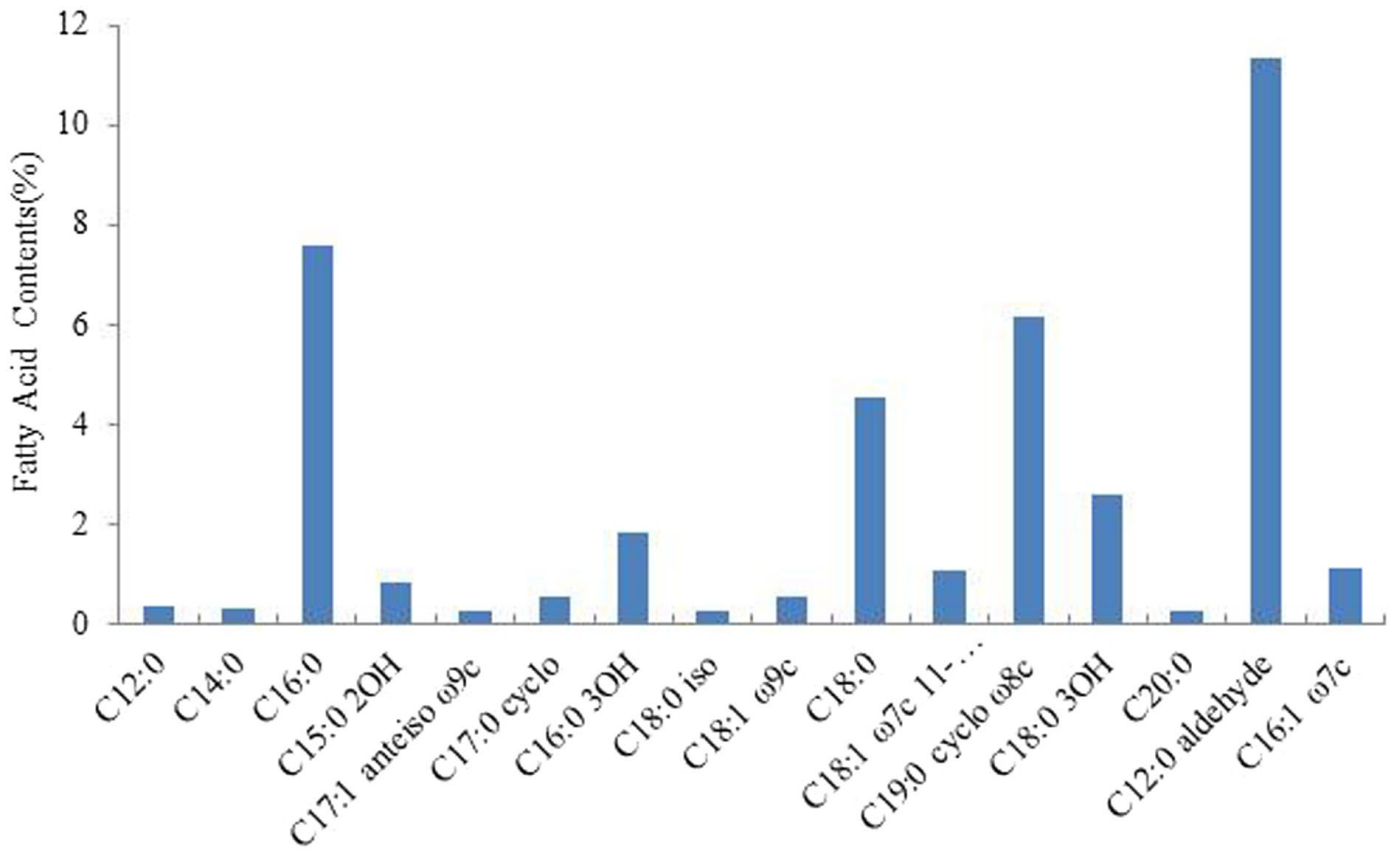
The fatty acid contents of *Rhizobium* ‘QHCD11’.

We analyzed the fatty acid content of *Rhizobium* ‘QHCD11’ and found that the cells contain C16:0 (7.58%), C19:0 cyclo ω8c (6.18%), C18:0 (4.54%), C18:0 3OH (2.61%), 12:0 aldehyde (summed feature 2, 11.37%), and 16:1 ω7c/16:1 ω6c (summed feature 3, 1.14%) (Figure 3). Similar fatty acid profiles were observed in the *Rhizobium* reference strains.

### Drought stress tolerance of the *Rhizobium* isolate

**Figure 4:**
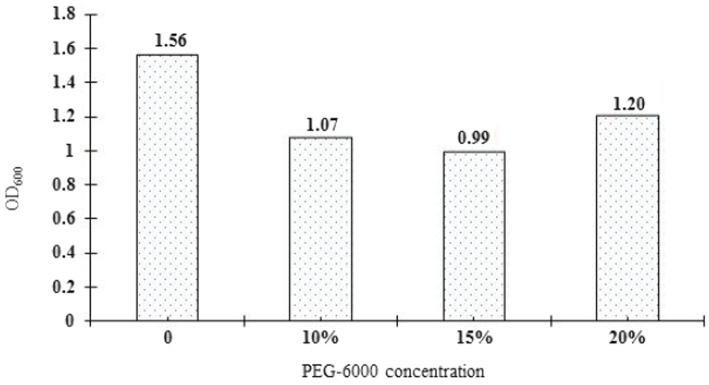
*Rhizobium* ‘QHCD11’ growth under drought-stressed conditions.

The drought tolerance of *Rhizobium* ‘QHCD11’ was evaluated by culturing the cells in YMA liquid culture media containing 0, 10, 15%, or 20% PEG-6000. The growth of the isolate, as determined spectrophotometrically based on OD_600nm_, was compared with *R. leguminosarum* reference strain ‘ACCC15854’ (Table 4). Increasing concentrations of PEG 6000 tended to result in decreased population size and optical density. Interestingly, *Rhizobium* ‘QHCD11’ performed better at 20% PEG 6000 than at 15% PEG 6000 (Figure 4), with the turbidity rising from 63.53 to 68.63%. Overall, *Rhizobium* ‘QHCD11’ was found to have superior drought tolerance. Research suggests that drought tolerant *Rhizobium* can improved the drought tolerance of host plants, so *Rhizobium* ‘QHCD11’ was selected for further studies of inoculation efficiency and growth improvement in faba bean under drought stress.

**Table 4:** Turbidity (*OD*_600nm_) of the rhizobium strains under simulated drought conditions.

Strain	*OD*_600nm_ at different PEG 6000 concentrations							Relative Turbidity (%)		
	0	10%	Decrement (%)	15%	Decrement (%)	20%	Decrement (%)	10%	15%	20%
QHCD11	1.56	1.07	5.77	0.99	36.47	1.07	31.37	93.90	63.53	68.63
ACCC15854	2.10	1.66	20.85	1.22	41.83	0.90	57.03	79.28	58.17	43.34

### Inoculation on faba bean growth under drought stress

Inoculation of plants with beneficial microbes has been shown to ameliorate abiotic stress (Egamberdieva et al., 2016). Here, we studied the biochemical, physiological, and morphological effects of inoculation with *Rhizobium* ‘QHCD11’ on ‘Qingcan 14’ faba bean seedlings subjected to drought stress. The results were compared with an uninoculated negative control and a positive control inoculated with *R. leguminosarum* reference strain ‘ACCC15854’ (Table 5).

**Table 5:** Morphophysiological indices of inoculated and uninoculated faba bean seedlings under control and drought-stressed conditions.

Treatment		Plant height (cm)	Nodules per plant	Fresh root nodule weight (g)	Fresh plant weight (g)	Root length (mm)	SPAD	RWC (%)	PRO (μg.g^-1^)	SOD (U·mg^-1^)	TTC [mg/(g.h)]
NN	CK	78.00ab	0.00	0.34cd	16.47c	42.40c	78.48ab	67.15de	535.93b	0.0028b	78.00ab
	DS	67.23b	0.00	0.21d	14.13c	35.52d	59.80c	51.29e	301.30c	0.0014b	67.23b
QHCD11	CK	85.30a	40.00a	0.68a	21.60b	55.67a	86.58a	118.82b	682.00a	0.0086a	85.30a
	DS	78.20ab	30.33b	0.51b	17.33c	50.11b	87.11a	151.91a	637.41a	0.0037b	78.20ab
ACCC 15854	CK	80.00ab	31.67ab	0.51b	28.64a	49.15b	82.94a	89.60cd	621.99ab	0.0081a	80.00ab
	DS	72.77ab	27.00 b	0.43bc	18.08bc	43.86c	70.19b	98.48bc	650.36a	0.0027b	72.77ab
LSD (*p*<0.05)		15.57	8.80	0.14	3.98	3.91	9.30	28.83	89.30	0.0024	15.57
ANOVA		4.11	15.00	22.74^*^	19.47	60.30^*^	17.33	33.19^*^	42.07	15.63	4.11

SPAD, SPAD values; RWC, Relative water content; PRO, Proline content; SOD, Superoxide dismutase activity; TTC, Triphenyltetrazolium chloride test; CK, Control (well-watered); DS, Drought stress (PEG-6000); NA, Inoculated; NN, Non-inoculated. Within the same column, different lowercase letters indicate significant differences according to the ANOVA test followed by the post-hoc Fisher test (LSD) with the 95% confidence interval.

Overall, inoculated faba beans (NA) exhibited substantially different characteristics than uninoculated faba beans (NN), with NA seedlings exhibiting improved drought resistance compared to NN seedlings. Unstressed inoculated seedlings exhibited the maximum for all tested parameters. Compared with matched controls, uninoculated faba bean seedlings were 13.8% shorter, had 59.52% fewer root nodules, and had 31.67% shorter roots (*p* ≤ 0.05). Unstressed *Rhizobium* ‘QHCD11’- inoculated seedlings were taller (85.3 cm) than unstressed uninoculated seedlings (78.0 cm). Drought-stressed *Rhizobium* ‘QHCD11’-inoculated seedlings had a greater number of root nodules (30.33) and a higher fresh root nodule weight (0.51 g) than uninoculated seedlings (11.33 and 0.21 g, respectively).

Physiological analyses revealed that drought stress not only hindered the biosynthesis of chlorophyll, but also accelerated the decomposition of existing chlorophyll. *Rhizobium* inoculation improved the SPAD value under both well-watered (+32.31%) and drought-stressed (+41.69%) conditions. Leaf RWC is an indicator of water status and reflects the balance between water supply and transpiration rate. Inoculation with *Rhizobium* ‘QHCD11’ significantly increased RWC by 81.05% under drought-stressed conditions and 48.05% under well-watered conditions.

Drought stress increased the proline content of faba bean seedlings, with *Rhizobium* ‘QHCD11’- inoculated seedlings exhibiting the greatest increase in proline. Compared to uninoculated seedlings, rhizobial inoculation resulted in a 38.14% increase in proline content under drought-stressed conditions. Furthermore, drought stress upregulated the activity of the enzymatic antioxidant SOD compared to well-watered conditions. Compared to un-inoculated seedlings, rhizobial inoculation increased SOD activity by 27.26% under well-watered conditions and a 33.76% increase in SOD activity under drought-stressed conditions. In water-limited environments, root health and vitality are predictors of survival (Zhang et al., 2022; Yu et al., 2023). The TTC test is an indicator of plant vitality broadly. Inoculated seedlings had significantly higher root vitality than uninoculated seedlings, especially *Rhizobium* ‘QHCD11’ -inoculated seedlings. Drought stress reduced root vitality, which was ameliorated with rhizobial inoculation. Increased root viability in inoculated plants is likely to have contributed to the increased drought tolerance of inoculated faba bean seedlings.

## Discussion

Crop yield can be severely impacted by abiotic stress, such as high temperatures, alkalinity, high salt, and drought. Both naturally occurring and inoculated microbes have proven efficacious in allaying abiotic stress in crops and other plants. Specifically, *Rhizobium* inoculants enhance plant abiotic stress resistance by decreasing the generation of ROS, improving leaf water status, facilitating water uptake, and maintaining ionic balance (Thrall et al., 2008). Native rhizobia collected from targeted host plants and coexisting leguminous plants can serve as resources for the development of new and improved crop inocula. Here, we characterized a rhizobial isolate collected from the roots of a faba bean plant growing in the arid Haidong City region in Qinghai Province, China. The isolate, ‘QHCD11’, exhibited a fast growth rate, acid production, and a morphological appearance in line with other members of the genus *Rhizobium*.

The ‘QHCD11’ 16S rRNA sequence was 1,477 bp long, and the draft genome was 7.50 Mbp in size, with a G + C content of 60.9%. Analysis of sequence identity indicated that the strain shared 99.93% similarity with *R. laguerreae* ‘FB206’^T^, *R. leguminosarum* ‘USDA 2370’^T^, *R. anhuiense* ‘CCBAU 23252’^T^, *R. ruizarguesonis* ‘UMP1133’^T^, and *R. indicum* ‘JKLM12A2’^T^. NJ phylogenetic analysis revealed that ‘QHCD11’ formed a phyletic group with *R. changzhiense* ‘WYCCWR 11279’^T^ (MH778807). Furthermore, phylogenomic analysis indicated that ‘QHCD11’ clustered with *R. indicum* ‘MCC3961’^T^. Our results were largely in agreement with previous studies suggesting that variation between Rhizobial strains may be more common at the species level, with diversity observed genus-wide. The ANI comparison value of strain ‘QHCD11’ to the closely-related *R. indicum* ‘FA23’^T^ was 95.62%, which is greater than the ≥95% threshold to discriminate between different species of microbes (Chun et al., 2018). To confirm the taxonomic status, genome sequence-based DDH was also utilized. The DDH value was 69.7%, close to the DDH threshold value of 70% (Meier-Kolthoff et al., 2013), indicating that strain ‘QHCD11’ belonged to the *R. indicum* ‘MCC 3961’^T^ species. Strain ‘QHCD11’ was found to contain a fatty acid array similar to *Rhizobium* reference strains, including18:1 ω7c (summed feature 8, 60.19%), 16:1 ω7c/16:1 ω6c (summed feature 3, 1.14%), 12:0 aldehyde (summed feature 2, 11.37%), C18:0 3OH (2.61%), C18:0 (4.54%), C19:0 cyclo ω8c (6.18%), and C16:0 (7.58%). These results clearly demarcate ‘QHCD11’ as a species of *R. indicum* (Richter and Rosselló-Móra, 2009). The Biolog GENIII bacterial identification system showed that strain ‘QHCD11’ was able to utilize several carbon sources, including tetrazolium blue, tetrazolium violet, acidum chinicum, glucuronamide, glucopyrone, L-glutamic acid, L-arginine, glycyl-L-proline, D-fructose-6-PO_4_, D-glucose-6-PO_4_, myo-phaseomannite, L-trehalose, D-galactose, N-Acetyl-D-galactosamine, N-Acetyl-β-D-mannosamine, D-meliodisaccharide, and D-honey trisaccharide. Based on these results, strain ‘QHCD11’ was preliminarily identified as belonging to the fast-growing genus *Rhizobium*.

Leguminous are important components of natural ecosystems and agricultural systems, and their microsymbionts of legumes have been intensively characterized (Han et al., 2010; Furseth et al., 2012; Zaw et al., 2021). Abiotic stresses are stress conditions to plants arising from the environment, it not only affected plants but also affect microbes. Many studies have shown that strains with drought tolerance can be screened out in arid environment, and inoculation of rhizobia can improve the growth of crops under abiotic stress conditions, increases crop yield and stress resistance (Enebe and Babalola, 2018; Bouhnik et al., 2022). Strain QHCD11 was identified by 16 s rRNA and whole genome sequencing as *Rhizobium indicum* species. Rahi et al. (2020) study found that *Rhizobium indicum* sp. nov., were characterized using 16S rRNA, atpD and recA genes, they were isolated from root nodules of pea (*Pisum sativum*) cultivated in the Indian trans-Himalayas. Earlier studies have reported that *Rhizobium* confers defence, enhances nodule number and promotes growth biomarkers in mung bean and *Vicia faba* (Ahmad et al., 2013; Benidire et al., 2017) under salt stress. Hussain et al. (2016) found a significant increase in the photosynthesis of maize plant as a result of inoculation with *Rhizobium phaseoli* under drought stress and confer tolerance to abiotic stresses. In this study, we isolated a strain from root nodueles of faba bean growing in the arid Qinghai province of China. We evaluated the drought tolerance of strain ‘QHCD11’ by subjecting the microbial culture to different concentrations of PEG-6000, and comparing the results to the reference *R. leguminosarum* strain ‘ACCC15854’. It shown that strain ‘QHCD11’ exhibited better drought tolerance in higher PEG osmotic solution and better than ‘ACCC15854’. Our findings are in line with the results of Bouhnik et al. (2022), who found the ability of the rhizobial strains isolated from the arid zones to grow in high concentrations of PEG-6000 could probably be due to adaptation to their original soils conditions.

Some researchers point out that inoculation of seeds with *Rhizobium* improved abiotic stress tolerance, it is attributed to produce light molecular weight organic solutes such as glycine betaine, proline, polyamines and ectoines, and these solutes protect the plant cells by stabilizing the structure and conformation of proteins as well as cell membranes from water stress, desiccation; increase photosynthetic and biomass productivity and also play role in the enhancement of the activity of various enzymatic and non-enzymatic antioxidants (Santos and Costa, 2002; Yurgel et al., 2013). In this study, we found that inoculation with strain ‘QHCD11’ initiates a wide response in various physiological activities like improves leaf water status, decreases the generation of reactive oxygen species, increase photosynthetic, enhance of the activity of various enzymatic antioxidants. *Rhizobium* inoculation improved the SPAD value under both drought-stressed and well-watered conditions. Furthermore, rhizobial inoculation can maintain leaf RWC by reducing dehydration-related cell damage, such as by increasing the proline content, as shown here. Enzymatic antioxidants like SOD alleviate oxidative stressed by converting ROS into O_2_ and water (Hasanuzzaman et al., 2012). Here, we found that inoculation with strain ‘QHCD11’ resulted in increased SOD activity, resulting in reduced oxidative stress and oxidative damage. And the same time, inoculated seedlings exhibited the best growth of all experimental groups under adequate water supply condition.

## Conclusion

In the present study, we characterized a fast-growing *Rhizobium* isolate (‘QHCD11’) which can utilize a range of carbon sources. Based on phylogenetic, chemotaxonomic, genotypic, and phenotypic analyses, the ‘QHCD11’ strain putatively belongs to the *R. indicum* ‘MCC 3961’^T^ species. Strain ‘QHCD11’ exhibited superior drought tolerance, and inoculation with this strain conferred drought tolerance and growth enhancement to faba bean seedlings. We suggest that *Rhizobium* strain ‘QHCD11’ may be further developed into a useful inoculant for improving the drought tolerance of faba beans and, potentially, other leguminous. These data help to illuminate the complex relationship between rhizobia and legumes under stressful environmental conditions.

## Data availability statement

The original contributions presented in the study are included in the article/Supplementary materials, further inquiries can be directed to the corresponding author. The data presented in the study are deposited in the NCBI repository, accession number SUB13746821 Seq OR412372.

## Author contributions

PL and TH designed experiments and wrote the manuscript. CT and JZ carried out experiments. CT and XW analyzed experimental results. YL analyzed sequencing data. All authors contributed to the article and approved the submitted version.

## Funding

This work was supported by the Qinghai Provincial Department of Science and Technology Project (2020-ZJ-709).

## Conflict of interest

The authors declare that the research was conducted in the absence of any commercial or financial relationships that could be construed as a potential conflict of interest.

## Publisher’s note

All claims expressed in this article are solely those of the authors and do not necessarily represent those of their affiliated organizations, or those of the publisher, the editors and the reviewers. Any product that may be evaluated in this article, or claim that may be made by its manufacturer, is not guaranteed or endorsed by the publisher.

## Footnotes

^1^http://www.ezbiocloud.net/eztaxon/

^2^http://jspecies.ribohost.com/jspeciesws/#home

^3^http://ggdc.dsmz.de/distcalc2.php

## Abbreviations

ANI: Average nucleotide identity
dDDH: Digital DNA–DNA hybridization
NJ: Neighbor-joining
YMA: Yeast mannitol agar
